# Baicalin inhibits IL-1β-induced ferroptosis in human osteoarthritis chondrocytes by activating Nrf-2 signaling pathway

**DOI:** 10.1186/s13018-023-04483-0

**Published:** 2024-01-03

**Authors:** Jiuxiang Liu, Hao Zhou, Jiangqi Chen, Qiang Zuo, Feng Liu

**Affiliations:** Department of Orthopedics, The First Affiliated Hospital of Nanjing Medical University (Jiangsu Province Hospital), No. 300 Guangzhou Road, Nanjing, 210029 Jiangsu Province China

**Keywords:** Baicalin, Osteoarthritis, Chondrocytes, Ferroptosis, Nuclear factor E2-related factor-2

## Abstract

**Background:**

Osteoarthritis (OA) is a common degenerative disease involving articular cartilage, in which ferroptosis of chondrocytes plays an important role. Baicalin (BAI) exerts regulatory effects in a wide range of orthopedic diseases including OA, but its effect on ferroptosis of chondrocytes (CHs) is still unclear. The purpose of this study was to determine the effect of BAI on ferroptosis in human OA chondrocytes (OACs), and to explore its possible mechanism.

**Methods:**

CHs were treated with IL-1β (10 ng/mL) to simulate inflammation in vitro. Immunofluorescence, quantitative RT-PCR, Western blotting and cell viability assay were performed to evaluate the impacts of BAI on Fe^2+^ level, mitochondrial dysfunction, ferroptosis-related proteins, oxidative stress and cytotoxicity in CHs. Additionally, siRNA was made use of to knock out nuclear factor E2-related factor 2 (Nrf2) to analyze the role played by Nrf2 in BAI-induced CH ferroptosis.

**Results:**

BAI eliminated IL-1β-induced Fe^2+^ accumulation, changes in mitochondrial membrane potential and ferroptosis-related protein GPX4, SLC7A11, P53 and ACSL4 levels, as well as reactive oxygen species (ROS), lipid peroxidation (LPO) and malondialdehyde (MDA) accumulation in CHs. Besides, BAI reversed IL-1β-induced decrease of Collagen II and increase of MMP13 in CHs. Meanwhile, BAI attenuated IL-1β-induced CH toxicity and promoted Nrf2 antioxidant system activation. When Nrf2 was knocked down by siRNA, the effects of BAI on IL-1β-induced ferroptosis-related proteins and antioxidant stress in CHs were significantly weakened.

**Conclusions:**

This study demonstrates that IL-1β can induce CH ferroptosis. BAI is able to inhibit IL-1β-induced CH ferroptosis and ECM degradation, and the specific mechanism may be that it can inhibit IL-1β-induced CH ferroptosis by activating Nrf2 antioxidant system to attenuate the accumulation of intracellular ROS and lipid ROS.

## Introduction

Osteoarthritis (OA), a chronic degenerative disease caused by joint strain, trauma, inflammation and other factors, is pathologically featured by articular cartilage degeneration, hardening and persistent inflammation, with the predilection sites being knee, hip, lumbar and other active joints [1]. In OA, the degeneration of articular cartilage that is composed of chondrocytes (CHs) and extracellular matrix (ECM) is closely related to abnormal CH damage and ECM degradation [2, 3]. Meanwhile, the synthesis/catabolism balance of CHs is also essential to maintain the integrity of ECM [4]. In 2012, Dixon et al. [5] reported for the first time an iron-dependent non-apoptotic cell death characterized by excessive accumulation of intracellular lipid reactive oxygen species (ROS), which is obviously different from the cell death mechanisms such as autophagy, apoptosis and necrosis at the morphological, metabolic and genetic levels. Intracellular iron homeostasis is precisely regulated by the iron metabolism system, and the maintenance of intracellular iron homeostasis is essential for cell function and fate [6]. The mechanism of ferroptosis, which has been found in a variety of tumors [7, 8] and degenerative diseases, is also shown to be significantly associated with OA progression [9, 10]. Previous studies have found that chondrocytes have various features of ferroptosis under pathological conditions: abnormal iron metabolism, lipid peroxidation, and mitochondrial dysfunction [11–13]. In addition, ferroptosis inhibitor ferrostatin-1 and iron ion chelator DFO have been found to delay the progression of OA in mice by inhibiting chondrocyte ferroptosis induced by iron overload environment [14, 15]. Therefore, it is of great significance to find potential drugs that target the inhibition of ferroptosis in chondrocytes for the treatment of OA.

Baicalin (BAI) is a natural flavonoid with broad anti-inflammatory, anti-tumor, and antioxidant actions. Duan et al. found that baicalin alleviated the pathological process of intracerebral hemorrhage in mice, and it inhibited the death of primary cortical neurons by blocking the iron-dependent cell death process, thereby alleviating motor deficits and brain damage in mice [16]. Fan et al. found that baicalin inhibited the expression of ACSL4, continuously blocked thee co-activation of TfR1 and NCOA4 mediated ferritin autophagy in cardiomyocytes, inhibited the accumulation of iron ions in myocardial tissue, and prevented myocardial ischemia/reperfusion injury in mice [17]. In addition, research has shown [18] that BAI can effectively ameliorate IL-1β-induced CH apoptosis and ECM degradation, and play an active role in the progression of OA. This study investigated the important characteristics of CH ferroptosis by using IL-1β to induce CHs to mimic inflammation, aiming to understand the regulatory action and possible mechanism of BAI on CH ferroptosis, and to determine whether BAI is protective of OA chondrocytes (OACs).

## Participants and methods

### Research participants

The cartilage tissues of 12 OA patients (5 males and 7 females that aged 58–69) who underwent total knee arthroplasty in the First Affiliated Hospital of Nanjing Medical University between July 2020 and March 2021 were collected to obtain human primary CH strains. All OA patients met the radiologic and clinical diagnostic criteria for OA revised by the American College of Rheumatology in 1995, and voluntarily signed an informed consent form. The human tissue experiments and methods involved in this study conform to the moral and ethical principles promulgated in the *Declaration of Helsinki*, and the research scheme has been reviewed and approved by the Institutional Ethics Committee of the First Affiliated Hospital of Nanjing Medical University.

### Cell treatment

(1) Cell separation: the articular cartilage obtained in total knee arthroplasty was rinsed with D-Hank's solution, cut into 1 mm^3^ slices and digested on an oscillator for 1 h at 37 °C with 0.2% collagenase II. Then, 0.25% trypsin was added to digest for 30 min. The digested cells were screened with a 120-mesh nylon net, and CHs were obtained after low-speed centrifugation to remove impurities. (2) Cell culture: the obtained human CHs were cultured in 10% FBS, 100U/mL penicillin and 0.1 mg/mL streptomycin supplemented DMEM according to the cell inoculation density of 1 × 10^5^/mL. (3) Cell treatment: according to the experimental requirements, human CHs were treated with 10 ng/mL IL-1β for 12 h or pretreated with 20 μM/mL BAI for 4 h, and then co-cultured with 10 ng/mL IL-1β and 20 μM/mL BAI for 12 h. (4) Cell transfection: small interfering RNA (siRNA) that specifically interferes with Nrf2 gene and negative control siRNA-NC were synthesized by Shanghai Sangon Biotech. Following the manufacturer's instructions, Nrf2 gene-targeting siRNA and negative control siRNA-NC were transfected into CHs by Lipofectamine 3000 (Invitrogen Company, USA) and cultured for 24 h. The Nrf2 gene sequence is as follows: 5′-CGACAGACCCTCCATCTA-3′.

### Cell viability assay

Human CHs were seeded at a cell density of 1 × 10^5^ cells/mL, inoculated with 100 μl of cells per well for 24 h, and intervened by IL-1β and BAI according to the grouping requirements. After treatment, CCK-8 reagent was added at 10 μl/well for 1 h of cultivation maintained at 37 °C. Cell viability was evaluated by measuring the absorbance value using a Multiskan FC microplate reader (Thermo Scientific, USA).

### Intracellular Fe^2+^ level detection

The Fe^2+^ level in CHs was determined by calcein-AM staining. CHs were incubated at 37 °C for 30 min with 1 mol/L calcein-AM. The fluorescence intensity of unchelated Fe^2+^ calcein was observed and measured under the magnification of fluorescence microscope (× 100 times) (excitation wavelength: 488 nm; emission wavelength: 517 nm), and three fields of view were randomly selected to determine the fluorescence intensity level.

### Intracellular mitochondrial membrane potential measurement

After the CHs were cultured and treated, they were washed with PBS three times. The mitochondrial membrane potential changes of CHs were measured using a JC-1 mitochondrial membrane potential detection kit (Solarbio, Beijing). The fluorescence changes and intensity of JC-1 monomers and multimers in CHs were detected using a DMi8 Leica fluorescence microscope (Leica, Germany).

### Detection of intracellular ROS, lipid peroxidation (LPO) and malondialdehyde (MDA) levels

CHs were treated with 3 PBS rinses after low-speed (1000 g) centrifugation for 10 min. Intracellular ROS and LPO were measured. After 20–30 min of light tight incubation in 10 μM DCFH-DA or 5 μm BODIPY-C11, cells were collected by centrifugation and immersed in PBS for 2 rinses. The collected cells were detected by a fluorescence microscope, and ROS and LPO levels were calculated by measuring fluorescence intensity. According to the instructions of the kit, the level of LPO (malondialdehyde, MDA) in CHs was measured by the LPO assay kit (Beyotime, Beijing), and the cellular MDA level was assessed by measuring the absorbance value of the reactants using a Multiskan FC microplate reader (Thermo Scientific, USA).

### Immunofluorescence staining

CHs (1 × 10^5^) seeded in 24-well plates were treated with IL-1β and BAI, respectively, immobilized with 4% paraformaldehyde at an ambient temperature for 15 min, and immersed in PBS for three rinses. Following this, they were permeated with 0.5%Triton X-100 for 20 min, and sealed with 5% BSA at room temperature for 1 h after washing with PBS buffer. After blocking, GPX4 primary antibody was added to incubate at 4 °C overnight. The next day, Cy3 labeled goat anti-rabbit antibody was added for light tight incubation for 1 h. Then came three PBS washes and the subsequent addition of nuclear dye DAPI for 5 min of culture. GPX4 fluorescence staining images in CHs were obtained with an LSM 900 laser confocal microscope (Carl Zeiss, Germany).

### RT-PCR

After being isolated from CHs using the TRIzol reagent (Invitrogen, USA), total RNA samples were subjected to purity and concentration determination by Nano Drop 2000 (Thermo Fisher Scientific, USA). The mRNA was reverse transcribed to prepare cDNA with retrovirus Primer Script™ kit (Takara, Dalian). Using a SYBR Premix Ex Taq II (Takara, Dalian) kit, quantitative PCR was carried out on ABI 7500 real-time fluorescence quantitative PCR instrument (ABI, USA). The target gene’s mRNA expression, normalized with U6, was calculated based on the 2^−ΔΔCt^ method. All operations were conducted in accordance with the instructions of the corresponding kit or instrument.

### Western blotting (WB)

CHs were inoculated in 6-well culture plates and treated differently after monolayer culture for 24 h. RIPA lysis buffer (Boster Company, China) was used to lyse and extract the cell samples, and the lysed extract was centrifuged (12,000 × *g*, 4 °C) for 30 min. The protein supernatant was collected and the protein concentration of the protein samples was detected by BCA analysis kit (Boster Company, China). After being diluted to an appropriate concentration, the protein sample was subjected to SDS-PAGE gel electrophoresis. Upon the completion of electrophoresis, the protein bands were transferred to a PVDF membrane (Millipore Company, USA) for 1 h of room temperature sealing with 5% skim milk and the subsequent overnight incubation with the specific primary antibodies against Col II, MMP13, MMMP3, Bax, NRF2, HO-1, Bcl-2 and β-actin maintained at 4 °C, followed by room temperature sealing with a secondary antibody for 1 h. The protein bands were reacted with ECL chemiluminescence substrate kit, and then imaged with gel imaging system (Bio-Rad Company, USA). The protein bands’ gray values were measured with ImageJ software, and the target proteins’ relative expression levels were calculated by the gray value ratio of the target protein/internal reference protein.

## Results

### BAI can alleviate IL-1β-induced CH toxicity

**Fig. 1:**
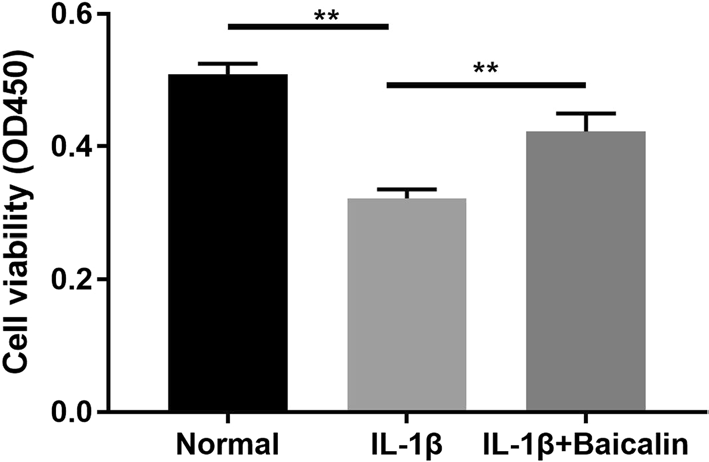
Baicalin reduced IL-1 β-induced chondrocyte activity. Chondrocytes were treated with IL-1β, and with or without baicalin for 12 h, determination of cell viability using the Cell Counting Kit-8 assay. *P < 0.05; **P < 0.01

The results of CCK-8 assay showed that IL-1β (10 ng/mL) significantly inhibited the cell viability of CHs cultured for 12 h compared with the blank control group (*P* < 0.05). However, BAI (20 μM) treatment contributed to markedly enhanced cell viability compared with IL-1β group (*P* < 0.05), which indicated that BAI could alleviate the effects of IL-1β induction on CH viability, as shown in Fig. 1.

### BAI can inhibit IL-1β-induced increase of Fe^2+^ level in CHs

**Fig. 2:**
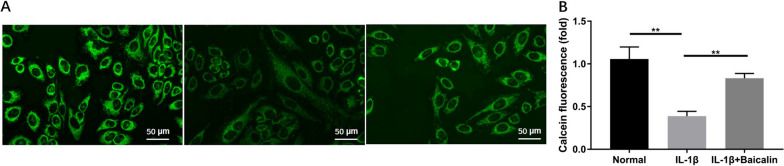
Baicalin reduced IL-1 β-induced cellular iron concentration. Chondrocytes were stained with a Calcein-AM fluorescent probe for 30 min after treatment. Observed by confocal microscopy. **A** Calcein fluorescence represents intracellular iron levels, and quenching of calcein fluorescence determines iron uptake by chondrocytes. The relevant data was shown in (**B**). *P < 0.05; **P < 0.01

Fe^2+^ levels in CHs were assessed by calcein-AM staining because intracellular Fe^2+^ chelates calcein to quench calcein fluorescence. The results exhibited that compared with the blank control group, the fluorescence intensity of calcein in CHs of IL-1β group decreased obviously, indicating obviously increased intracellular Fe^2+^ level (*P* < 0.05). However, IL-1β + BAI co-culture led to notably enhanced calcein fluorescence intensity compared with the IL-1β group, suggesting significantly decreased intracellular Fe^2+^ level (*P* < 0.05). It is suggested that IL-1β can induce the increase of Fe^2+^ level in CHs, while BAI can reduce Il-1β-induced Fe^2+^ accumulation in CHs, as shown in Fig. 2.

### BAI can inhibit IL-1β-induced mitochondrial dysfunction in CHs

**Fig. 3:**
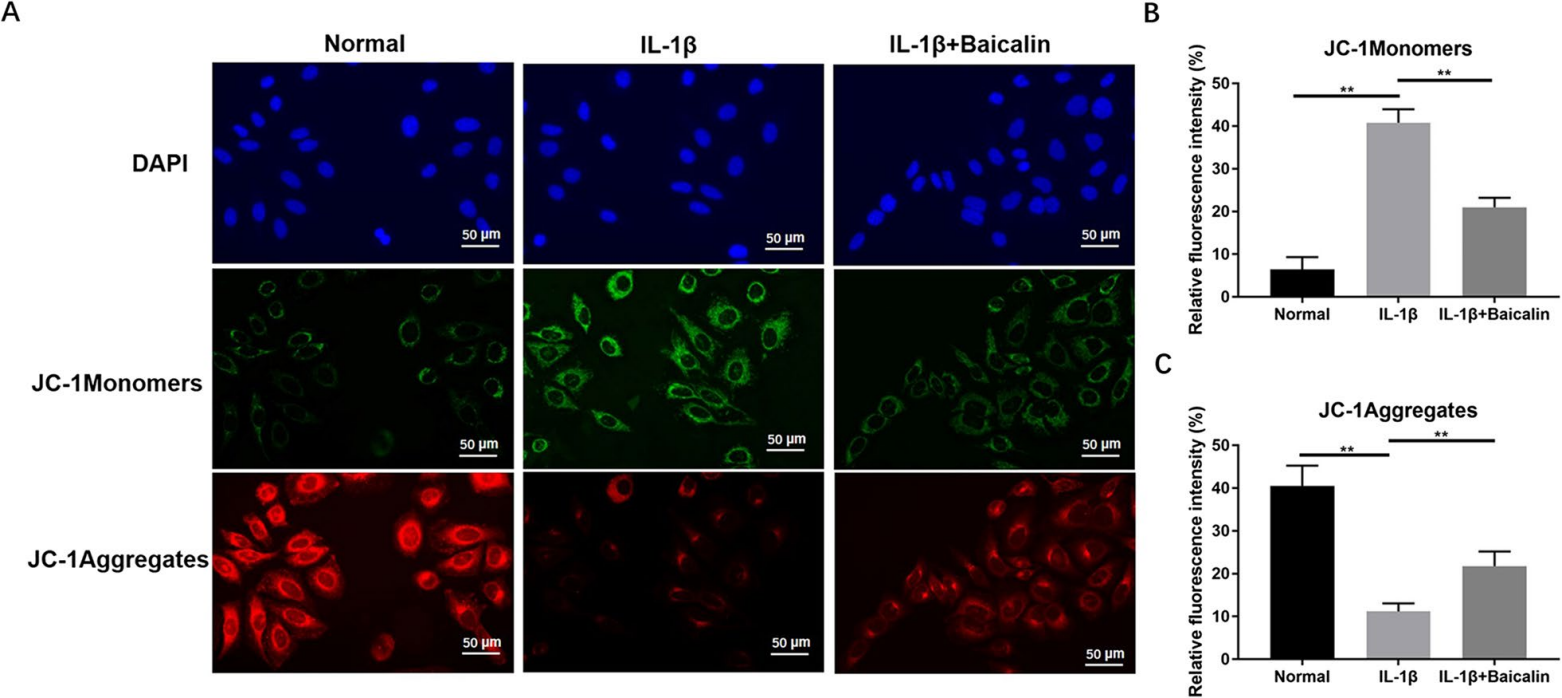
Baicalin reduced IL-1 β-induced mitochondrial membrane potential. **A** Chondrocytes were stained with a JC-1 fluorescent probe for 15 min after treatment. Observed by confocal microscopy. The relevant data was shown in (**B** and **C**). *P < 0.05; **P < 0.01

Ferroptosis mainly leads to mitochondrial dysfunction by reducing the mitochondrial membrane potential of cells. This study used JC-1 staining to detect changes in mitochondrial membrane potential. The results identified that in the CHs of the blank control group, JC-1 existed in the form of multimers in mitochondria and excited red fluorescence. In contrast, after IL-1β treatment, the mitochondrial transmembrane potential was depolarized, and JC-1 was released from the mitochondria at a reduced concentration, which reversed it to the green fluorescence-emitting monomeric form. While BAI reversed the change of fluorescence intensity of CHs treated with IL-1β, with obviously increased red fluorescence. These results suggest that BAI inhibits IL-1β-induced changes in mitochondrial membrane potential of CHs and reverses mitochondrial dysfunction caused by ferroptosis, as shown in Fig. 3.

### BAI reverses IL-1β-induced expression of ferroptosis-related protein markers in CHs

**Fig. 4:**
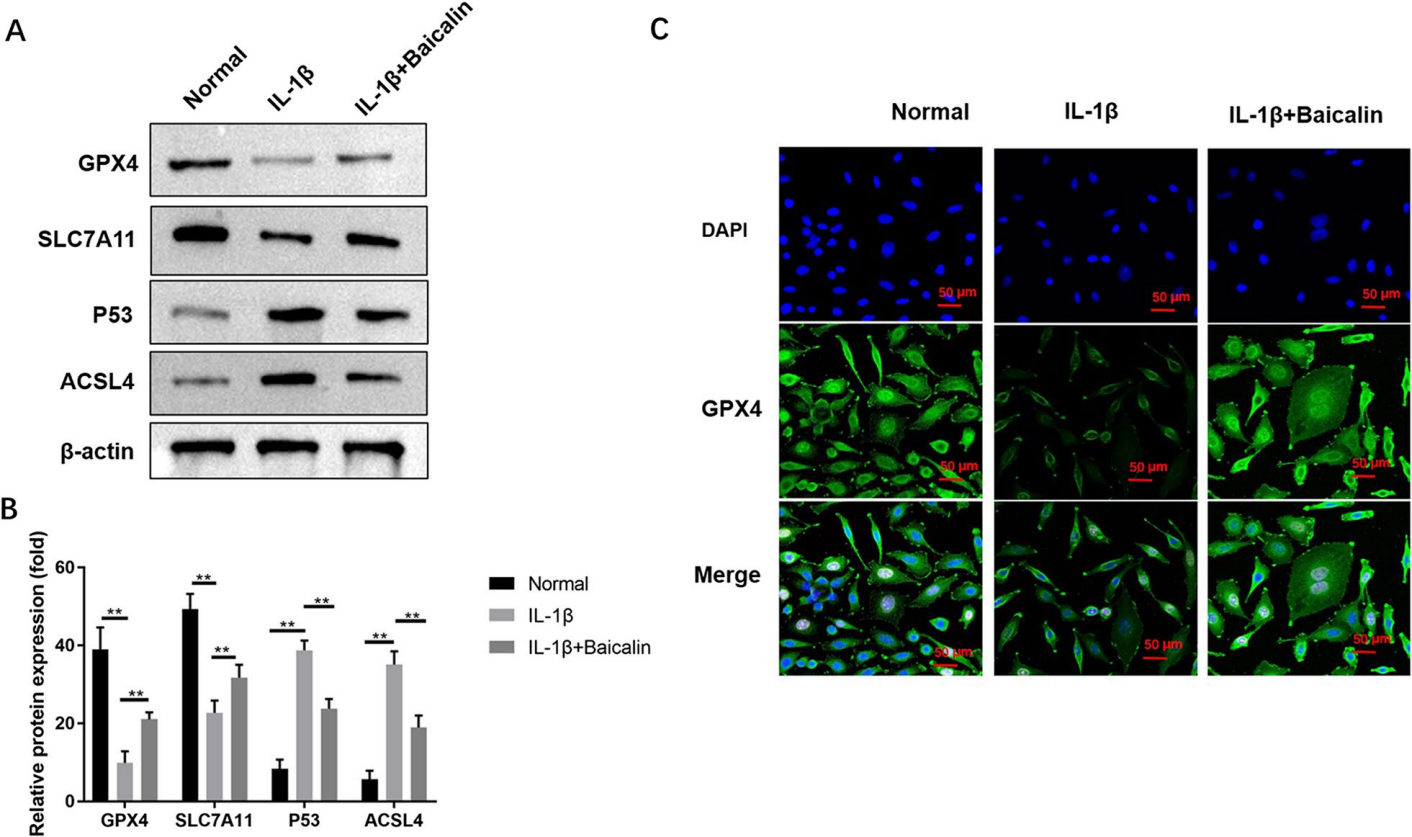
Baicalin inhibited IL-1 β-induced ferroptosis-related protein expression changes in chondrocytes. **A** The expression levels of GPX4, SLC7A11, P53, ACSL4 were detected by Western blot. The relevant data was shown in (**B**). The total GPX4 protein level in the chondrocytes was evaluated by immunofluorescence staining (**C**). *P < 0.05; **P < 0.01

WB was used to detect ferroptosis-related protein markers (Fig. 4). It was found that the levels of ferroptosis-related proteins GPX4 and SLC7A11 in CHs in the IL-1β group dropped statistically compared with the blank control group, while P53 and ACSL4 increased significantly (*P* < 0.05). BAI reversed the IL-1β-induced changes in the expression levels of ferroptosis-related proteins in CHs (*P* < 0.05).

**Fig. 5:**
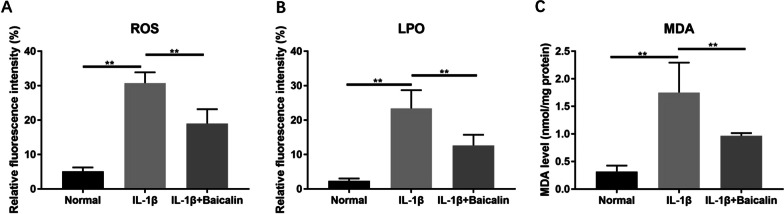
Baicalin reduced IL-1 β-induced ROS and LPO accumulation. Chondrocytes were stained with DCFH-DA or C11 BODIPY fluorescent probe, ROS and LPO levels were calculated by measuring fluorescence intensity (**A** and **B**). The absorbance of oxidation reaction was measured to evaluate the cellular MDA level by using kits (**C**). *P < 0.05; **P < 0.01

The expression and distribution of GPX4, a key protein of ferroptosis, in CHs were further detected by immunofluorescence staining. The results showed a large amount of cytoplasmic GPX4 protein in CHs of the blank control group. After IL-1β (10 ng/mL) treatment for 48 h, the uniform and dispersed fluorescent particles of cytoplasmic GPX4 protein in CHs of the IL-1β group decreased obviously, and the fluorescence intensity decreased statistically (*P* < 0.05). The BAI + IL-1β group exhibited significantly enhanced fluorescence intensity of GPX4 protein than the IL-1β group (*P* < 0.05). See Fig. 5 for details.

### BAI can inhibit the levels of ROS, LPO and MDA in CHs induced by IL-1β

**Fig. 6:**
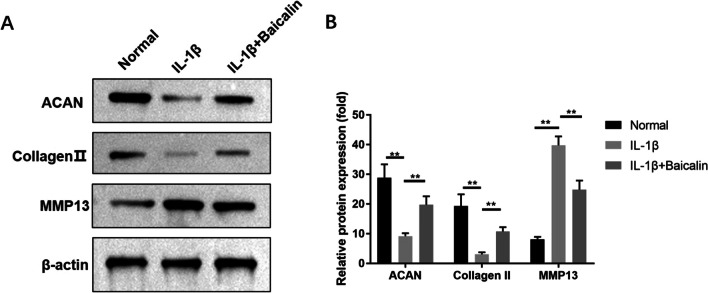
Baicalin attenuated IL-1 β-induced cartilage degradation and increased the Collagen II and ACAN expression in chondrocytes. **A** The expression levels of MMP13, Collagen II and ACAN were detected by Western blot. The relevant data was shown in (**B**). *P < 0.05; **P < 0.01

The accumulation of ROS, LPO and MDA in cartilage cells is also an important feature of ferroptosis. The results showed significantly increased levels of ROS, LPO and MDA in CHs after IL-1β (10 ng/mL) treatment (*P* < 0.05), while BAI reduced IL-1β-induced ROS, LPO and MDA accumulation (*P* < 0.05), as shown in Fig. 6.

### BAI can inhibit IL-1 β-induced ECM degradation in CHs

**Fig. 7:**
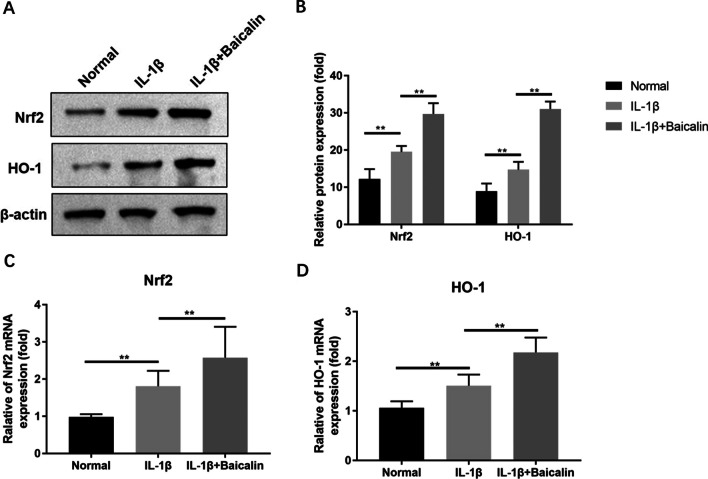
Baicalin promoted the expression levels of Nrf2 and its downstream factors in chondrocytes. **A** The expression levels of Nrf2 and HO-1 were detected by Western blot. The relevant data was shown in (**B**). The mRNA expression levels of Nrf2 (**C**) and HO-1 (**D**) were detected by RT-PCR. *P < 0.05; **P < 0.01

Collagen II is the main component of CH ECM, while MMP13 is the key enzyme for ECM degradation. The decrease of Collagen II secretion and the significant overexpression of MMP13 in CH are the hallmarks of OA. WB analysis showed that MMP13 protein in CHs treated with IL-1β was significantly over-expressed compared with the blank control group, while Collagen II expression level was markedly inhibited *(P* < 0.05); however, this process was significantly reversed by BAI treatment (*P* < 0.05). The above results suggest that BAI can inhibit IL-1β-induced ECM degradation in CHs. See Fig. 7 for details.

### BAI inhibits IL-1β-induced CH ferroptosis by activating Nrf2 signaling pathway

**Fig. 8:**
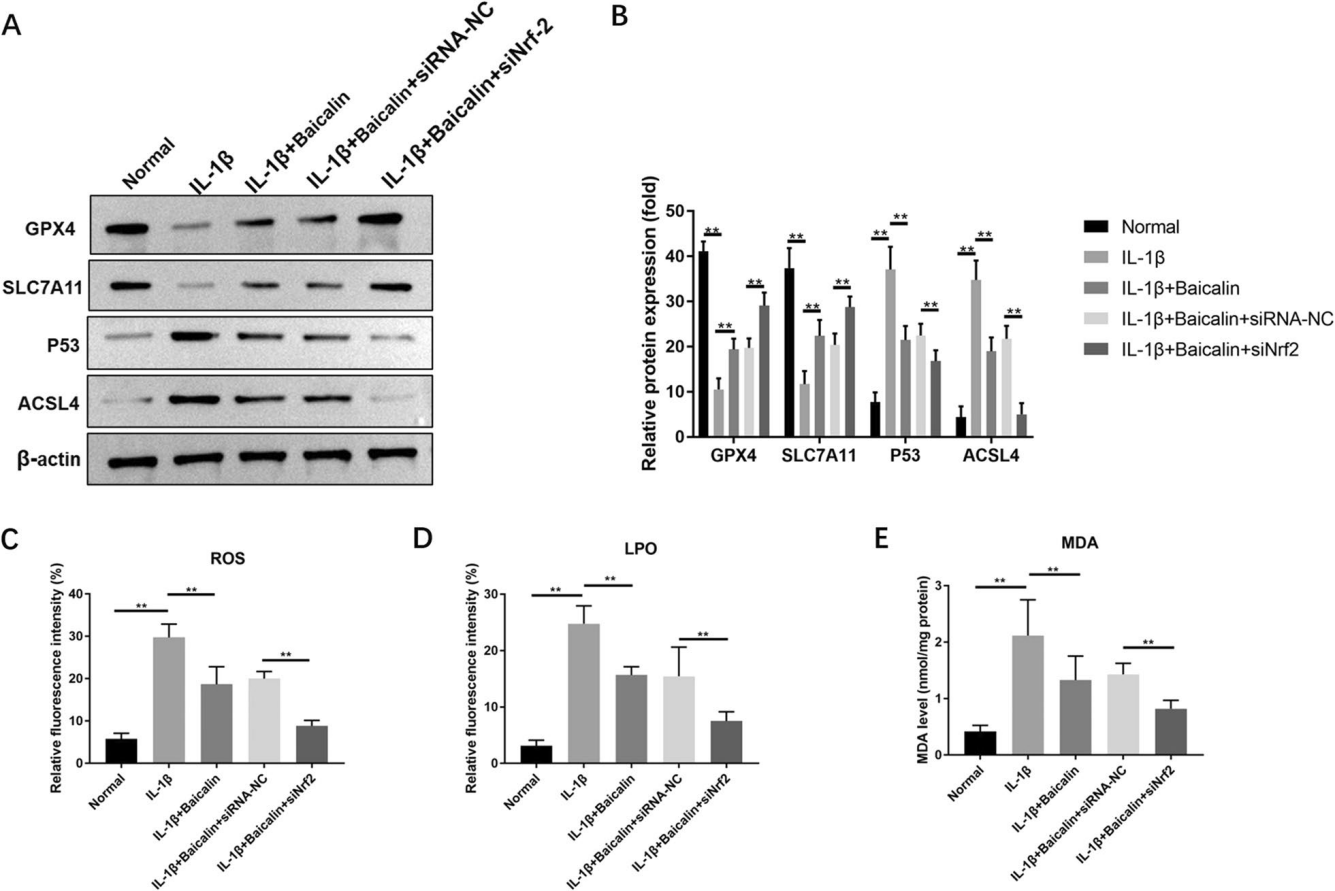
Baicalin inhibited IL-1β-induced ferroptosis in chondrocytes by activating Nrf2 signaling pathway. Chondrocytes were treated with IL-1β, and with or without baicalin for 24 h, and then the Nrf2 signaling pathway was blocked by transfection of siNrf2. **A** The expression levels of GPX4, SLC7A11, P53, ACSL4 were detected by Western blot. The relevant data was shown in (**B**). Baicalin reduced IL-1β-induced ROS (**C**), LPO (**D**) and MDA (**E**) accumulation by activating Nrf2 signaling pathway. *P < 0.05; **P < 0.01

In this study, WB analysis showed that IL-1β (10 ng/mL) treatment promoted the transcription and protein expression of Nrf2 protein and its downstream effector HO-1, while BAI (20 μM) + IL-1β (10 ng/mL) intervention further enhanced the effect, suggesting that BAI activated the Nrf2 antioxidant system in CHs. Further, we silenced the transcriptional expression of Nrf2 in CHs by siRNA in the BAI + IL-1β group. It was found that Nrf2 knockdown reduced the effect of BAI on the transcription and expression levels of ferroptosis-related proteins GPX4, SLC7A11, P53 and ACSL4 in CHs (*P* < 0.05), while promoting ROS, LPO and MDA accumulation (*P* < 0.05). Based on the above results, it is speculated that the Nrf2 axis is essential in the inhibition of IL-1β-induced CH ferroptosis by BAI, and BAI may play a role in inhibiting IL-1β-induced CH ferroptosis by activating the antioxidant system of Nrf2 to increase the antioxidant level. See Fig. 8 for details.

## Discussion

Ferrum is an essential trace element to maintain the normal function of the human body, and one of its important functions is to become a catalyst for various biological reactions in the body and participate in various biochemical metabolic reactions based on the powerful redox properties of Fe^2+^. However, a large amount of intracellular iron accumulation will generate excessive ROS-induced cellular oxidative stress (OS) damage, causing multiple toxic effects on cells and eventually leading to cellular ferroptosis [19]. Ferroptosis is a new controllable programmed cell death mode characterized by iron-dependent accumulation of intracellular ROS and lipid ROS, leading to mitochondrial membrane thickening, ridge reduction and cell membrane fragmentation. A series of abnormal gene expression and signal transduction are involved in the process of programmed cell death [20–22]. With the in-depth research on ferroptosis and degenerative diseases in recent years, more and more evidence has demonstrated the close connection between ferroptosis and OA progression.

OA is a degenerative disease mainly featured by articular cartilage degeneration, and CH ferroptosis plays a key role in the occurrence and development of OA. Simão et al. [23] showed that iron accumulation, caused by abnormal iron metabolism-induced multiple cytotoxicity to various musculoskeletal cells, may be one of the key influencing factors of OA. An early study [24] tested various metal trace elements in synovial fluid of patients with rheumatoid arthritis and OA, and found significantly higher iron concentration in the synovial fluid of such patients than in healthy people. Jing et al. [25] reported that iron overload may aggravate cartilage degradation and bone loss of subchondral bone, with serum ferritin level positively correlated with the degree of joint injury. In this study, the results of in vitro experiments on human OACs showed that IL-1β could induce Fe^2+^ elevation, mitochondrial dysfunction, intracellular ROS, LPO and MDA accumulation, and other important features of ferroptosis in human OACs. In addition, IL-1β induced decreases in ferroptosis-related proteins GPX4 and SLC7A11 in CHs, and significant increases in P53 and ACSL4 protein levels. Furthermore, IL-1β intervention-induced CH ECM degradation and cytotoxic effects. All these results suggest that IL-1β can induce CH ferroptosis and cause CH damage. Yao et al. [15] further revealed that both IL-1β and iron overload could induce ROS and LPO accumulation in mouse CHs and change the expression of iron death-related proteins in CHs, in addition to decreasing type II collagen secretion and increasing MMP13 expression. While Ferrostatin-1, an ferroptosis inhibitor, can effectively block this process, which shows that CHs wound undergo ferroptosis under inflammatory conditions and that ferroptosis promotes OA-like changes such as inflammation, OS and ECM degradation in CHs. Thus, inhibition of ferroptosis in CHs may become the main research direction to prevent OA, bringing new hope to patients.

Previous studies have shown [26, 27] that BAI can provide protection to CHs of patients with OA by regulating the expression of various genes and signaling pathways, which reflects its potential clinical application value in preventing and treating OA. However, the role played by BAI in CH ferroptosis remains elusive. The accumulation of ROS caused by iron overload is the key mechanism that induces iron-dependent programmed cell death. However, Pan et al. [28] reported that BAI could significantly inhibit H2O2-induced OS in endplate CHs, reduce MDA production, and increase superoxide dismutase (SOD) and nitric oxide (NO) levels, thus effectively preventing endplate CH apoptosis. It is suggested that BAI, which has a broad anti-OS effect, may play an active role in ROS accumulation caused by iron overload. The results of this study confirmed that BAI could obviously inhibit the important characteristics of ferroptosis in CHs induced by IL-1β, such as the increase of Fe^2+^, mitochondrial damage, and the accumulation of intracellular ROS, LPO and MDA. And the results of WB showed that BAI also increased the levels of ferroptosis-related regulatory proteins GPX4 and SLC7A11, and inhibited P53 and ACSL4 protein expression, reflecting the effective inhibitory effect of BAI on CH ferroptosis. Moreover, BAI could partially reverse the cytotoxic damage of IL-1β to CHs, effectively increase the production of collagen II, the main component of CH ECM, and reduce the expression of MMP13, a key ECM degrading enzyme. Therefore, BAI can alleviate IL-1β-induced CH ferroptosis and protect CHs against ferroptosis-induced cell matrix destruction and cytotoxicity.

Nrf2 is a member of the bZip family of transcription factors, and the Nrf2 axis is the main antioxidant system in cells. Multiple studies have found [29–31] that inhibition of Nrf2 and its downstream effectors in various tumor cells and nerve cells leads to obvious features of ferroptosis, suggesting that Nrf2 is a key factor in alleviating cellular LPO and ferroptosis. Guo et al. [32] showed that deferoxamine, a ferroptosis inhibitor, could effectively inhibit mouse CH ferroptosis induced by IL-1β or iron overload, and promote the over-expression and nuclear translocation of Nrf2 and its downstream effector HO-1. In addition, Nrf2 knock-out partially abolished the inhibition of IL-1β-induced CH ferroptosis by deferoxamine. These findings suggest that ferroptosis may block the Nrf2 axis in response to OS, while deferoxamine activates Nrf2 and its downstream effectors to act against OS and inhibit the occurrence of ferroptosis in CHs. Pan et al. [33] also showed that naringenin inhibited the OS of CHs by activating NRF2-HO-1 pathway, thus alleviating ferroptosis characteristics such as intracellular ROS accumulation, membrane LPO and mitochondrial dysfunction caused by IL-1β and iron overload, and finally achieving the purpose of protecting CHs and preventing OA. The results of this study revealed the ability of BAI to effectively promote the overexpression of Nrf2 and HO-1 in human OACs induced by IL-1β; however, after knockdown of Nrf2 by siRNA, the ability of BAI to protect CHs from IL-1β-induced ferroptosis was obviously weakened. These results suggest that IL-1β-induced CH ferroptosis is partially realized by influencing the Nrf2 axis, and the Nrf2-mediated antioxidant system is essential to protect CHs from ferroptosis. BAI may improve the antioxidant level by activating the Nrf2 antioxidant system, thus inhibiting IL-1β-induced CH ferroptosis and cytotoxicity.

To sum up, this paper argues that BAI can protect human OACs from IL-1β-induced ferroptosis, ECM degradation and cytotoxicity. Furthermore, the inhibition of IL-1β-induced ferroptosis by BAI may be achieved by activating the NRF2-HO-1 pathway, thereby inhibiting OS in CHs. These results provide certain references for the research and development of new drugs for the prevention and treatment of OA.

## Data Availability

The data used for this study have been included in the manuscript.
